# Copper Complexes of Nicotinic-Aromatic Carboxylic Acids as Superoxide Dismutase Mimetics

**DOI:** 10.3390/molecules13123040

**Published:** 2008-12-08

**Authors:** Thummaruk Suksrichavalit, Supaluk Prachayasittikul, Theeraphon Piacham, Chartchalerm Isarankura-Na-Ayudhya, Chanin Nantasenamat, Virapong Prachayasittikul

**Affiliations:** 1 Department of Clinical Microbiology, Faculty of Medical Technology, Mahidol University, Bangkok 10700, Thailand; E-mails: rukmu47@gmail.com (T. S.); mttpc@mahidol.ac.th (T. P.); mtcis@mahidol.ac.th (C. I.); mtcnt@mahidol.ac.th (C. N.); mtvpr@mahidol.ac.th (V. P.); 2 Department of Chemistry, Faculty of Science, Srinakharinwirot University, Bangkok 10110, Thailand; E-mail: supaluk@swu.ac.th (S. P.)

**Keywords:** Nicotinic acid, Copper, Carboxylic acid, Superoxide dismutase, Antimicrobial activity

## Abstract

Nicotinic acid (also known as vitamin B3) is a dietary element essential for physiological and antihyperlipidemic functions. This study reports the synthesis of novel mixed ligand complexes of copper with nicotinic and other select carboxylic acids (phthalic, salicylic and anthranilic acids). The tested copper complexes exhibited superoxide dismutase (SOD) mimetic activity and antimicrobial activity against *Bacillus subtilis* ATCC 6633, with a minimum inhibition concentration of 256 µg/mL. Copper complex of nicotinic-phthalic acids (CuNA/Ph) was the most potent with a SOD mimetic activity of IC_50_ 34.42 µM. The SOD activities were observed to correlate well with the theoretical parameters as calculated using density functional theory (DFT) at the B3LYP/LANL2DZ level of theory. Interestingly, the SOD activity of the copper complex CuNA/Ph was positively correlated with the electron affinity (EA) value. The two quantum chemical parameters, highest occupied molecular orbital (HOMO) and lowest unoccupied molecular orbital (LUMO), were shown to be appropriate for understanding the mechanism of the metal complexes as their calculated energies show good correlation with the SOD activity. Moreover, copper complex with the highest SOD activity were shown to possess the lowest HOMO energy. These findings demonstrate a great potential for the development of value-added metallovitamin-based therapeutics.

## Introduction

Nicotinic acid (NA) or vitamin B3 is essential for many biological processes namely for the production of energy [1], signal transduction, regulation of gene expression [2] and involvement in the synthetic pathway of lipids [3]. The oxidation of lipoproteins, low density lipoprotein and very low density lipoprotein, by free radical, particularly superoxide radical (O_2_^•-^), causes vascular inflammation, which is involved in the early stage development of atherosclerosis, a disease affecting arterial blood vessels [4]. In developed countries such as the United States, atherosclerosis is the leading cause of illness and death accounting for 44% of deaths and morbidity [5]. Nicotinic acid has been reported to decrease the production of free fatty acids and lipoproteins [6], despite of this free radical continues to appear in the human body. To eliminate such superoxide radicals, transition metal complexes were developed as superoxide dismutase mimics. Some examples include metalloporphyrin [7] and metal-drug complexes [8].

Complexation of nicotinic acid with various metals, e.g. manganese, cobalt, nickel, copper and zinc had been previously reported [9]. Nicotinic acid-copper complex (CuNA) has been shown to exert diverse bioactivities. In particular, it can stimulate blood flow and prevent gastric congestion [10], reduce total lipids in sera hepatic tissue as well as regulate levels of alanine transaminase, aspartate transaminase, alkaline phosphatase, gamma glutamyl transpeptidase and oxidative markers such as nitric oxide (NO) and lipid peroxidation in rat models [11]. Additionally, it also exhibits SOD mimic activity in patients with hepatocellular carcinoma. The metal complexes of nicotinic acid with other small molecules such as isonicotinate and acetylacetonate were previously reported [9,12]. The aforementioned nicotinic acid-copper complexes were formed through coordination with the carboxylate oxygen- and/or pyridine nitrogen-atoms [12,13], which are electron donors. These electron-donating groups are commonly found in many natural compounds such as flavonoids (rutin, taxifolin, epicatechin and luteolin), phenolics (catechol and resveratrol) and drugs (aspirin, ibuprofen and oxaprozin), all of which were reported to form metal complexes with potent superoxide scavenging capacities [14,15,16]. For example, flavonoid copper complexes were shown to exhibit higher SOD activity than the parent free flavonoids [15]. A series of simple Cu carboxylate (acetate, salicylate or benzoate) complexes synthesized by Devereux *et al*., were reported to exhibit excellent SOD mimic activity [17]. Salicylic acid, one of the metal-chelating ligands used in this study, is a natural compound found in most fruits and vegetables and functioning as a plant growth hormone. The compound was shown to possess promising anti-cancer activity by modulating the inhibition of the P form of phenolsulphotransferase, thus preventing excessive carcinogen activation [18]. Anthranilic acid (also known as vitamin L), another ligand used in the study, is a natural ingredient essential for lactation. Its derivatives have been found to activate soluble guanylyl cyclase, a ubiquitous NO receptor involved in vasorelaxation and hypertension [19]. In addition, copper atom is an essential nutrient involved in the catalytic function of many enzymes such as copper-zinc superoxide dismutase (CuZnSOD) [20,21]. Copper deficiency has been reported to cause hematologic disorders, hypopigmentation, defective connective tissue cross-linking and ataxia [22,23].

The ideal SOD-mimic should be a low molecular weight metal complex that possesses high membrane permeability. However, very few Cu-carboxylate complexes had been reported [17]. In an attempt to obtain small molecule-based metal complexes, we now report the synthesis of novel copper complexes as potent SOD mimics. Such complexes are based on the coordination of nicotinic acid-Cu with the small molecule carboxylates phthalic acid (Ph), salicylic acid (Sal) and anthranilic acid (Ant). Computational chemistry has found extensive applications for studying molecular structure and reactivity [24]. Some usage examples include studying the mechanism of protein receptor-inhibitor interaction [25], development of quantitative structure-activity/property relationship (QSAR/QSPR) [26], and elucidation of enzymatic reaction [27], etc. To shed light on the underlying mechanisms of the superoxide radical scavenging activities, quantum chemical parameters were calculated at DFT level. Results indicated that the calculated physicochemical parameters were well correlated with the experimental SOD activities. Such theoretical parameters included energies of HOMO and LUMO as well as EA. Furthermore, to confer value-added benefits to the copper complexes as well as explore the possibility of using such compounds as a multifaceted drug the antimicrobial activity of the novel complexes was also determined.

## Results and Discussion

Three copper-based complexes, copper complexes of nicotinic-phthalic acids (CuNA/Ph, **1**), nicotinic-salicylic acids (CuNA/Sal, **2**) and nicotinic-anthranilic acids (CuNA/Ant, **3**), were synthesized by the reaction of 1:1:1 ratio of cupric chloride with nicotinic acid and aromatic carboxylic acids (phthalic, salicylic and anthranilic acids). The complexes were obtained in excellent yields (82-91%) as aquamarine powder, highly polar, insoluble in methanol and water, but soluble in dimethyl sulfoxide (DMSO). Their melting points (m.p.) and magnetic moments (µ_eff_) are listed in Table 1.

**Table 1:** Physicochemical parameters of nicotinic acid-copper complexes and ligands.

Compound	Chemical Formula	Formula Weight (g∙mol^-1^)	Color	Melting Point (°C)	Yield (%)	µ_eff_ (B.M.)
NA	C_6_H_5_NO_2_	123.11	white	236-239	−	−
Ph	C_8_H_6_O_4_	166.13	white	210	−	−
CuNA/Ph (**1**)	C_14_H_10_CuNO_7_	367.78	aquamarine	>300	82	1.6944
Sal	C_7_H_6_O_3_	138.12	white	158-160	−	−
CuNA/Sal (**2**)	C_13_H_10_CuNO_6_	339.77	aquamarine	>300	85	1.6802
Ant	C_7_H_7_NO_2_	137.14	yellow	144-148	−	−
CuNA/Ant (**3**)	C_13_H_13_CuN_2_O_5_	338.78	aquamarine	>300	91	1.8038

### Infrared spectra

**Figure 1:**
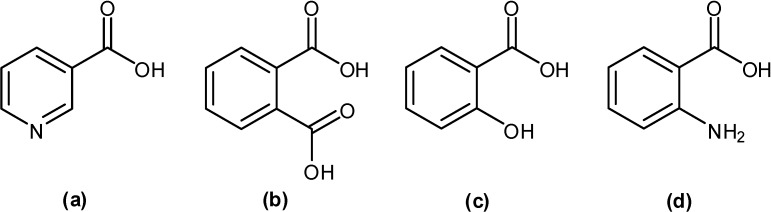
Molecular structure of ligands: nicotinic acid (a), phthalic acid (b), salicylic acid (c), and anthranilic acid (d).

Coordination of the copper atom with the functional groups of the ligands was established from the IR spectra (Table 2). From the data of complex **1**, it can be seen that the C-O stretching vibration frequency was located at 1,298 cm^-1^, while the free phthalic acid and nicotinic acid absorptions υ(C-O) appeared at 1,282 and 1,299 cm^-1^, respectively. In addition, disappearance of the hydroxyl group bending vibration (δO-H) of Ph at 1,404 cm^-1^ was observed. For the carbonyl group (C=O), the stretching vibrations of both NA and Ph still exhibited intensive bands at 1,685, 1,701, and 1,718 cm^-1^. The weak band at 1333 cm^-1^ could be ascribed to C-N stretching vibration of complex **1** as compared with the spectra of NA, which shows strong band υ(C-N) at 1324 cm^-1^. Thus, complex **1** was formed using pyridine ring nitrogen atom of NA and two hydroxyl groups of Ph to coordinate with copper atom. Structures of ligands are shown in Figure 1.

**Table 2:** IR spectra of the free ligands and the copper coordination complexes.

Cpd	υC=O	υC-O	υC-N	υO-H	δO-H	υNH	δNH
NA	1,718 (s)	1,299 (s)	1,324 (s)	3,072-2,449 (w)	1,418 (s)	−	−
	1,700 (s)						
Ph	1,700 (s)	1,282 (s)	−	3,072-2,524 (br)	1,404 (s)	−	−
	1,685 (s)						
**1**	1,718 (s)	1,298 (m)	1,333 (w)	3,080-2,565 (w)	1,418 (m)	−	−
	1,701 (s)						
	1,685 (s)						
Sal	1,662 (s)	1,296 (s)	−	3,471 (br)	1,446 (s)	−	−
	1,655 (s)	1,250 (s)		3,240 (sh)	1,484 (s)		
		1,211 (s)					
		1,211 (s)					
**2**	1,717 (s)	1,298 (s)	1,332 (m)	2,682 (sh, w))	1,454 (m)	−	−
	1,700 (s)	1,201 (w)		2,567 (sh, w)	1,420 (m)		
	1,685 (s)	1,144 (w)					
		1,126 (w)					
Ant	1,679 (m)	1,277 (m)	1,371 (s)	2,869 (br)	1,458 (m)	3,325 (sh, s)	754 (vs)
	1,654 (sh, m)	1,238 (m)	1,319 (s)	2,580 (br)		3,239 (sh, s)	
				2,362 (sh, w)			
				3,449 (br)			
**3**	1,718 (s)	1,298 (s)	1,386 (s)	3,400 (br)	1,458 (m)	3,275 (m)	756 (s)
	1,700 (s)		1,332 (m)	2,681 (w)	1,420 (m)		
				2,565 (w)			

Note: vs = very strong, s = strong, m = medium, w = weak, br = broad, sh = sharp.

In a similar fashion, the IR spectra of complex **2** were interpreted by comparison of its spectra with those of the free ligands. It was apparent that the hydroxyl and carbonyl absorptions of salicylic acid were at 3,471 cm^-1^ υ(O-H) and 1,655, 1,662 cm^-1^ υ(C=O), respectively, suggesting that Cu-complex formation took place via the phenolic and carbonyl functionalities. The IR spectra of complex **2** still displayed strong absorptions at 1,700, 1,717 cm^-1^ υ(C=O), 1,298 cm^-1^ υ(C-O) and 1,420 cm^-1^ δ(O-H). However, the C-N stretching vibration of complex **2** was observed at 1,332 cm^-1^ whereas the C-N absorption of free NA appeared at 1,324 cm^-1^. Based on the IR spectra, complex **2** was observed to be formed through the carbonyl and the phenolic hydroxyl group of Sal and the pyridine ring nitrogen atom.

The IR spectra of complex **3** exhibited disappearance of the anthranilic acid carbonyl (C=O) absorption at 1,654 and 1,679 cm^-1^. Additionally, the amino (NH) absorption of complex **3** appeared at 3,275 cm^-1^, while the NH_2_ absorption of Ant was observed at 3,239 and 3,325 cm^-1^. Strong C-N stretching of Ant was displayed at 1,371 and 1,319 cm^-1^ with a very strong NH bending observed at 754 cm^-1^. The IR spectra of complex **3** showed strong absorptions at 1,718, 1,700 (C=O), 1,298 (C-O), medium bending band of OH at 1,420 cm^-1^ and C-N stretching of ring N-atom at 1,332 cm^-1^. The characteristic free NA C-N stretching absorption of the pyridine ring (υ 1,324 cm^-1^) was shifted to higher frequency at 1332 cm^-1^. The C-N stretching of NH group of complex **3** was observed at 1,386 cm^-1^. It was suggested that complex **3** was formed through coordination of pyridine ring nitrogen atom; carbonyl and NH groups of Ant with Cu centered atom.

It is noted that these Cu-complexes were formed via monodentate interaction of the ring nitrogen atom of NA resulting in a υ(C-N) shift from 1,324 cm^-1^ to a higher frequency range of 1,332-1,333 cm^‑1^. The υ(C=O), υ(C-O) and δ(O-H) absorptions of the NA ligand of the complexes remained unperturbed. Aside from NA, other ligands coordinated with the central Cu atom via bidentate carboxylates. With regards to complex **1**, Ph is the bidentate ligand using two hydroxyls of dicarboxylic acid to coordinate with the Cu atom. This is observed by the disappearance of C-O stretching at 1,282 cm^-1^ and O-H bending at 1,404 cm^-1^. As for complex **2**, stretching at 1,662 and 1,655 cm^-1^ (C=O) and at 3,471 and 3,240 cm^-1^ (O-H) disappeared with a shift of C-O stretching from 1,250, 1,211 and 1,156 cm^-1^ to lower frequencies. This suggested that the Cu atom formed a complex by means of a bidentate phenolic carboxylate. Similarly, the Cu atom of complex **3** was found to be coordinated with the amino carboxylate of Ant ligand, which is due to the disappearance of C=O and NH_2_ stretching absorptions of Ant.

High resolution mass spectra (HRMS) measurements for complexes **1**-**3** were performed, but their molecular ions were not detected. Fragmented ions were observed at m/z of 124.0399 corresponding to free nicotinic acid as calculated for C_6_H_5_NO_2_ [M+H]^+^. Additionally, m/z of 124.0399 was quantified for complexes **1** and **2** while 124.0396 was detected for complex **3**.

Complexes **1-3** were paramagnetic, with magnetic moments (µ_eff_) of 1.69, 1.68 and 1.80 B.M., respectively. The µ_eff_ value indicates that the complexes were of tetrahedral conformation with the Cu(II) center. Therefore, on the basis of both IR spectra and µeff, we can confirm that complexes **1-3** were of tetrahedral geometry with the Cu(II) center.

### Superoxide scavenging activity

The copper complexes were assayed for SOD-like activity using the modified method by measuring the inhibition of the photoreduction of nitro blue tetrazolium (NBT). The results showed that all the tested complexes exhibited SOD activities with IC_50_ in the range of 34.42-47.49 µM as presented in Table 3.

**Table 3:** Superoxide dismutase activity of the free ligands and copper complexes.

Compound*	*IC_50_ (µM)^a^*
Sal	>594.00
Ant	>236.25
**1**	34.42
**2**	42.79
**3**	47.49
**SOD^b^**	0.0026

^a^ IC_50_ was defined as fifty percent inhibition concentration of NBT reduction. ^b^ Superoxide dismutase from bovine erythrocytes was a homodimeric protein. * Nicotinic acid and phthalic acid possessed very low activity, to the point that the IC_50_ cannot be obtained.

Complex **1** was the most potent SOD mimic with an IC_50_ of 34.42 µM. Moreover, the SOD activity of the free ligand, NA and Ph, was very low to the point that the IC_50_ could not be obtained. The SOD activity of the uncoordinated salicylic and anthranilic acids was assayed and their IC_50_ was determined to be 594 and 236.25 µM, respectively. It was observed that complex **2** was approximately 14 times more potent than the uncoordinated Sal. Similarly, complex **3** was five times more potent than the free Ant. The results support the notion that the copper atom is essential for SOD activity and that the incorporation of copper into the structure of the ligands tremendously increased the SOD activity. We attribute this to the change in oxidation state of the copper atom modulated through its coordination with the metal-chelating ligands. It is noteworthy that the coordination complexes are much smaller than the native enzymes. Moreover, an added benefit is that the compounds can be easily synthesized from simple bioactive compounds. Some of the potent SOD mimics developed thus far were based on Mn and Cu coordination compounds. Example of such SOD mimics are the Cu-drug complexes which included Cu (aspirin), Cu (aspirin)_4_ and Cu (ibuprofenate).

### Antimicrobial activity

Nicotinic acid is a widely used drug for the treatment of hyperlipidemia [3]. In addition, salicylic and anthranilic acids are bioactive aromatic carboxylic acids. The antimicrobial activity of complexes **1-3** has not been reported before. As the objective of this study is to develop value-added metallovitamins for therapeutic applications, therefore it is worthy to observe whether the compounds could also be used as antimicrobials, aside from being superoxide radical scavengers. Our results revealed that the tested complexes **1-3** can selectively inhibit the growth of *B. subtilis* ATCC 6633 with a MIC of 256 µg/mL (Table 4).

**Table 4:** Antimicrobial activity of the free ligands and nicotinic acid-copper complexes.

Compound	Concentration (µg/mL)	Activity
Ampicillin	25	Active^a^
NA	256	Not Active
CuCl_2_	64*	Not Active
	32*	Not Active
Ph	256	Not Active
**1**	256	Active^b^
	128	Active^c^
	64	Not Active
Sal	256	Active^b^
**2**	256	Active^b^
	128	Active^d^
	64	Not Active
Ant	256	Not Active
**3**	256	Active^b^
	128	Active^c^
	64	Not Active

^a^ Active (100% antigrowth) against *P. aeruginosa* ATCC 15442, *S. putrefaciens* ATCC 8671, *A. xylosoxidans* ATCC 2706, *S. aureus* ATCC 25923, *S. epidermidis* ATCC 12228, *E. faecalis* ATCC 29212, *B. subtilis* ATCC 6633, *S. cereviseae* ATCC 2601, *S. pyogenes* II, *S. enteritidis* type C, *P. shigelloides*, *L. monocytogenes*. Active against *B. subtilis* ATCC 6633 with ^b^ 100%, ^c^ 50%, ^d^ 25% antimicrobial activity. * CuCl_2_ was tested against *B. subtilis* ATCC 6633.

It should be noted that salicylic acid was the only free ligand that could completely inhibit the growth of *B. subtilis* ATCC 6633 (MIC of 256 µg/mL) and that the antimicrobial activity of salicylic acid and its derivatives against *E. coli*, *B. subtilis* and *S. aureus* has been previously reported [28]. Aside from this, the other free ligands (e.g. nicotinic, phthalic and anthranilic acids) were inactive against all of the tested microorganisms. A plausible explanation for the antimicrobial activity of the copper complexes is that they may enhance bacterial killing by synergistically converting superoxide radical to hydrogen peroxide in which accumulation of hydrogen peroxide exerted harmful effect to the bacterial cells as well as participated in the subsequent formation of hydroxyl radical via the Fenton’s reaction.

### Molecular modeling of SOD mimics

**Figure 2:**
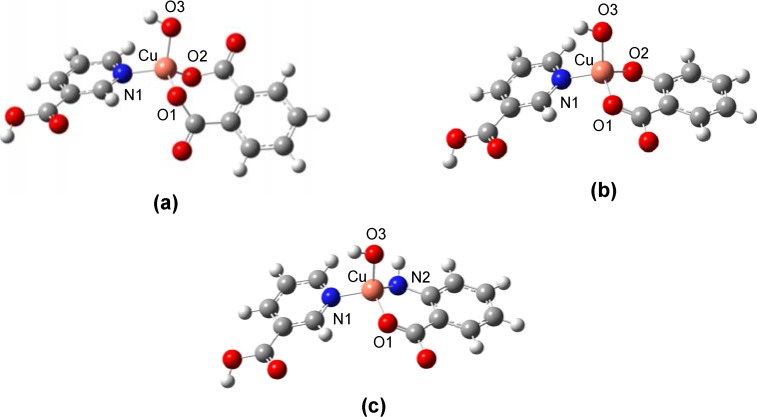
Molecular structures of copper coordination complexes **1** (a), **2** (b), and **3** (c).

To elucidate the mechanisms of radical scavenging activities of the copper complexes, density functional theory calculations at the B3LYP/LANL2DZ level was employed. Previous efforts have indicated that the calculation of theoretical parameters, binding energies and electron affinities, at such theoretical levels are suitable for characterizing the superoxide radical scavenging activity [29,30,31,32]. The molecular structures were constructed on the basis of IR and magnetic moment data which indicated that the coordination complex was of distorted tetrahedral conformation. Geometrically optimized structure of complexes **1-3** are presented in Figure 2.

Electron affinity, in particular, is an appropriate theoretical parameter accounting for the electron transfer rate from superoxide anion to copper atom [30,31,32]. The lower the EA becomes the higher the electron transfer rate is, which correspondingly leads to higher superoxide radical scavenging activity. EA was calculated according to equation (1) by taking the difference of the total energy of Cu(II) and Cu(I) coordination complexes. The EA for complexes **1**-**3** as presented in Table 5 were calculated to be -125.502, -139.283 and -212.679 kcal/mol, respectively.

**Table 5:** Theoretical parameters of the copper complexes.

Complex	TE_Cu(II)_^a^ (hartree)	TE_Cu(I)_^b^ (hartree)	EA* (kcal/mol)
**1**	-1316.673	-1316.873	-125.502
**2**	-1203.223	-1203.445	-139.283
**3**	-1183.292	-1183.631	-212.679

^a^ Total energy of the Cu(II) coordination complex ^b^ Total energy of the Cu(I) coordination complex * EA was calculated according to equation (1)

The experimental SOD activities of complexes **1-3** had an IC_50_ of 34.42, 42.79 and 47.49 µM, respectively. The results indicated that there exists a positive correlation between EA and SOD activity where high EA gives rise to high SOD activity, which is inversely correlated in previous reports [30,31]. This is presumably due to differences in the coordination geometry of the copper complexes used in this study (tetragonally distorted) and the copper complexes (distorted square-planar and square-pyramidal) reported previously [30,31]. Similar observation was deduced by Branco *et al*. in their studies on active site distortion of CuZnSOD [33,34].

Additionally, the usefulness of quantum chemical descriptors such as energy of the highest occupied molecular orbital and energy of the lowest unoccupied molecular orbital in elucidating the radical scavenging activity were also investigated. Examples on the usage of HOMO and LUMO energies in accounting for chemical reactivities of molecules and their relevance to electron transfer complexes can be found in an excellent review by Karelson and Lobanov [35]. The HOMO energies of the ligands were calculated to be -5.818, -6.729 and -7.644 eV, respectively with the following order: Ant > Sal > Ph (Table 6).

**Table 6:** HOMO and LUMO energies of the free ligands and copper complexes.

Compound	HOMO (eV)	LUMO (eV)
Ph	-7.644	-2.252
Sal	-6.729	-1.749
Ant	-5.818	-1.553
**1**	-7.302	-4.898
**2**	-6.293	-4.595
**3**	-5.829	-4.086

Likewise, the calculated value of HOMO for the complexes exhibited similar trend: **3** > **2** > **1** with the corresponding values of -5.829, -6.293 and -7.302 eV, respectively. It is well established that HOMO accounts for the electron donating ability while LUMO characterizes the ability to accept electron [36]. From the frontier molecular orbital approximation, high HOMO energy value infers that the molecule or ligand can easily release electrons to the unoccupied orbital of the metal ion, indicating strong binding affinity [37]. Thus, Ant possesses the strongest interaction with copper leading to the best binding capacity. Such degree of binding capacity is found to be Ant > Sal > Ph.

In addition, the selected bond lengths and angles of the geometrically optimized structures are given in Table 7. The average bond distance at axial position for Cu-N1 of complexes **1-3** was 2.029 Å. The longest axial bond length for Cu-O1 and Cu-O2 were observed for complex **1** with values of 1.872 and 1.870 Å, respectively. The axial bond lengths of Cu-O1 and Cu-O2 for complex **2** was found to be shorter than that of complex **1** with values of 1.838 and 1.857 Å, respectively. This is explained by the inductive effect of the two carbonyl groups of complex **1** withdraws electrons from the Cu atom giving rise to low electron donating ability of the ligand as also indicated by the lower HOMO energy. Therefore, complex **1** exhibited the highest SOD activity. Finally, the shortest axial bond lengths for Cu-N2 and Cu-O1 were found in complex **3** to be 1.809 and 1.830 Å, respectively. This can be attributed to the greater electron donating ability of the amino group of anthranilic acid than the phenolic group of salicylic acid. This results in the lower HOMO energy of **2** than **3**. It was previously reported by Li *et al*. [38] that axial bond lengths were crucial for SOD activity where long bond lengths being advantageous for the dismutation of superoxide anion. In addition, correlation between the binding capacity and the SOD activity of metal complexes was previously studied using molecular modeling and quantum chemical calculation [8]. It was found that molecules exhibiting higher metal binding affinity displayed lower SOD activity [8,39]. This was observed for complex **3** which possessed the lowest SOD activity and the highest calculated HOMO value. On the other hand, complex **1** exhibited the highest SOD activity with the lowest calculated HOMO value (Table 3 and Table 6). Furthermore, the calculated energies of HOMO and LUMO were well correlated with the SOD activity as observed from *r* = 0.999 and 0.953, respectively. Such results demonstrate the practical usage of HOMO and LUMO as theoretical parameters for the characterization of SOD activity in terms of charge- or electron-transfer of the complex.

**Table 7:** Selected bond distances (Å) and angles (°) of complexes **1-3**.

Complex	Bond Lengths (Å)		Angles (°)	
**1**	Cu-N1	2.035	N1-Cu-O1	111.475
	Cu-O1	1.872	N1-Cu-O2	111.235
	Cu-O2	1.870	N1-Cu-O3	107.711
	Cu-O3	1.829	O1-Cu-O2	105.661
			O1-Cu-O3	110.275
			O2-Cu-O3	110.513
**2**	Cu-N1	2.035	N(1)-Cu-O1	109.012
	Cu-O1	1.838	N(1)-Cu-O2	111.596
	Cu-O2	1.857	N(1)-Cu-O3	109.467
	Cu-O3	1.836	O(1)-Cu-O2	107.727
			O(1)-Cu-O3	109.929
			O(2)-Cu-O3	109.355
**3**	Cu-N1	2.016	N1-Cu-O1	109.926
	Cu-N2	1.809	N1-Cu-O2	109.365
	Cu-O1	1.830	N1-Cu-N2	110.023
	Cu-O2	1.835	N2-Cu-O2	109.091
			N2-Cu-O1	108.241
			O1-Cu-O2	109.581

## Conclusions

The novel copper complexes with nicotinic acid and carboxylic acids were synthesized in excellent yields. The coordination compounds were characterized to be tetragonally distorted structures formed by monodentate coordination of ring N-atom of NA as well as bidentate carboxylate ligands using amino carbonyl (Ant), phenolic carbonyl (Sal) and two hydroxyls (Ph). It should be noted that from the chemical and biological point of view, these copper complexes could easily be formed through the coordination of metal ions with simple dietary intake of vitamins or endogenous carboxylic acids and related compounds. All of the copper complexes were shown to exhibit superoxide scavenging activity as well as antimicrobial activities against *B. subtilis* ATCC 6633 with MIC 256 µg/mL. Complex **1** was the most potent SOD activity with an IC_50_ of 34.42 µM. Theoretical parameters as calculated by DFT methods were used to elucidate the mechanisms of SOD activity. Interestingly, copper complexes with higher SOD activity such as complex **1** were found to have higher EA value. Furthermore, calculated HOMO and LUMO energies displayed good correlation with the SOD activities with *r* of 0.999 and 0.953, respectively. Such results corroborate the usefulness of HOMO and LUMO energies in relation to the SOD activity as it could account for the charge- or electron-transfer of the complex. These findings suggest great potential for the development of metallovitamin-based therapeutics.

## Experimental

### General

Melting points of the complexes were determined on the Griffin capillary melting point apparatus and are reported uncorrected. Infrared (IR) spectra were obtained on a Perkin Elmer System 2000 FTIR using potassium bromide (KBr) pellet. Mass spectra were recorded on a Bruker Daltonics (MicroTOF) instrument. Magnetic moments were measured with a Mark 1 Magnetic Susceptibility Balance (Sherwood Scientific, Cambridge, UK). Nicotinic acid, phthalic acid, salicylic acid, and anthranilic acid were of analytical grade and purchased from Sigma-Aldrich, as was bovine erythrocyte superoxide dismutase.

### Synthesis of mixed-ligand complexes of copper with nicotinic acid and phtalic acid (1)

Nicotinic acid (0.123 g, 1 mmol) dissolved in methanol (30 mL) was heated (70°C) and stirred under reflux until a clear solution was obtained. Cupric chloride dihydrate (0.170 g, 1 mmol) dissolved in methanol (2 mL) and then added dropwise to the prepared nicotinic acid solution. After heating for 45 min, the reaction mixture was added dropwise to a solution of phthalic acid (1 mmol, 0.166 g) in methanol (2 mL), then, the mixture was heated for 1 h under the same conditions. The precipitated solid was collected by filtration, washed with cold methanol and dried under vacuum over silica gel at room temperature. Complex **1** was obtained as an aquamarine powder; m.p. > 300°C; IR (KBr, cm^-1^): 2,565-3,080 (m, OH), 1,685 (s, CO), 1,701 (s, CO), 1,718 (s, CO), 1,418 (m, OH), 1,333 (w, CN), 1,298 (m, C-O); HRMS (TOF) calculated for C_6_H_5_NO_2_ [M+H]^+^ 124.0399, found: 124.0399.

### Synthesis of mixed-ligand complexes of copper with nicotinic acid and salicylic acid (2)

Complex **2** was prepared in the same way as complex **1** using nicotinic acid (0.123 g, 1 mmol), cupric chloride (0.170 g, 1 mmol) and 1 mmol (0.138 g) of salicylic acid. Product **2** was obtained as an aquamarine powder by filtration, washing with cold methanol and dried over silica gel; m.p. > 300°C; IR (KBr, cm^-1^): 2,567 (sh, w, OH), 2,682 (sh, w, OH), 1,717 (s, CO), 1,700 (s, CO), 1,685 (s, CO), 1,454 (m, OH), 1,420 (m, OH), 1,332 (m, CN), 1,298 (s, C-O), 1,201 (w, C-O), 1,144 (w, C-O), 1,126 (w, C-O); HRMS (TOF) calculated for C_6_H_5_NO_2_ [M+H]^+^ 124.0399, found: 124.0399.

### Synthesis of mixed-ligand complexes of copper with nicotinic acid and anthranilic acid (3)

Complex **3** was prepared by an analogous procedure, but changing from Ph and/or Sal to anthranilic acid (1 mmol, 0.137 g) as a ligand. The precipitate of complex **3** was received as an aquamarine powder; m.p. > 300°C; IR (KBr, cm^-1^): 3,275 (m, NH), 3,400 (br, OH), 2,681 (w, OH), 2,565 (w, OH), 1,718 (s, CO), 1,700 (s, CO), 1,608 (s), 1,558 (vs), 1,458 (m, OH), 1,420 (m, OH), 1,386 (s, CN), 1,332 (m, CN), 1,298 (s, C-O); HRMS (TOF) calculated for C_6_H_5_NO_2_ [M+H]^+^ 124.0399, found: 124.0396.

### Determination of superoxide dismutase (SOD)-like activity

The complexes were tested for SOD activity using the previously described method [8]. The SOD activity of copper complexes was assayed by measuring the inhibition of the photoreduction of NBT. This indirect assay is comprised of several reactions: the photochemically excited riboflavin was first reduced by methionine to a semiquinone, which donated an electron to oxygen to form the superoxide source. The superoxide readily converted NBT into a purple formazan product, which was spectrophotometrically detected at 550 nm. In this regard, the SOD activity was inversely related to the amount of formazan formed, and expressed in term of IC_50_ of NBT reduction.

### Antimicrobial activity

The antimicrobial activity of the complexes was investigated against representative microorganisms using the method previously reported [40]. One milliliter of Müeller Hinton (MH) broth was mixed with the tested complexes dissolved in DMSO. The MH broth of the tested sample was then mixed with MH agar solution and placed onto the plates with the final concentrations (64, 128 and 256 μg/mL) as agar dilution. The microorganisms, cultured in the MH broth at 37 °C for 24 h, were diluted with 0.9% normal saline solution to 3×10^8^ cell/mL. The organisms were inoculated onto each plate and incubated at 37 °C for 18-48 h. Complexes found to be effective against the tested strains were selected for further investigations. The inhibition of microbial cell growths was also determined. Twenty-seven strains of microorganisms were used as shown in Table 8.

### Molecular modeling of superoxide dismutase mimics

The molecular models of the copper complexes were constructed with GaussView 3.09 [41] based on the IR and magnetic moment data which indicated that the coordination complexes were of distorted tetrahedral conformation. Full geometry optimizations without symmetry constraints were performed *in vacuo* using Becke’s three-parameter hybrid Lee-Yang-Parr (B3LYP) [42] functional and the LANL2DZ [43,44,45] basis set under Gaussian 03W [46]. The LANL2DZ basis set was selected as it could handle high Z atoms [47], particularly atoms that are beyond the third row.

Electron affinity was computed by taking the difference of the total energies of the coordination complexes Cu(II) and Cu(I) as summarized by the following equation:

*EA* = *TE_Cu(I)_* − *TE_Cu(II)_* (1)

where *EA* represents the electron affinity, *TE_Cu(I)_* represents the total energy of the Cu(I) coordination complex, *TE_Cu(II)_* represents the total energy of the Cu(II) coordination complex. HOMO and LUMO energies were derived from geometrically optimized Cu(I) coordination complex as it is the active form of the SOD mimetics.

**Table 8:** Organisms subjected to growth inhibition assays.

*Microorganism*	*Reference strain*	*Clinical isolate*
**Gram-positive bacteria**	*Staphylococcus aureus* ATCC 29213	*Streptococcus pyogenes* II
	*Staphylococcus aureus* ATCC 25923	*Bacillus cereus*
	*Staphylococcus epidermidis* ATCC 12228	*Listeria monocytogenes*
	*Enterococcus faecalis* ATCC 29212	
	*Enterococcus faecalis* ATCC 33186	
	*Micrococcus lutens* ATCC 10240	
	*Bacillus subtilis* ATCC 6633	
	*Corynebacterium diphtheriae* NCTC 10356	
**Gram-negative bacteria**	*Escherichia coli* ATCC 25922	*Shigella dysenteriae*
	*Klebsiella pneumoniae* ATCC 700603	*Salmonella enteritidis* type C
	*Serratia marcescens* ATCC 8100	*Morganella morganii*
	*Salmonella typhimurium* ATCC 13311	*Aeromonas hydrophila*
	*Shewanella putrefaciens* ATCC 8671	*Citrobacter freundii*
	*Achromobacter xylosoxidans* ATCC 2706	*Plesiomonas shigelloides*
	*Pseudomonas aeruginosa* ATCC 15442	
	*Pseudomonas stutzeri* ATCC 17587	
**Yeasts**	*Saccharomyces cereviseae* ATCC 2601	
	*Candida albicans* ATCC 90028	
